# Pilot study using an optical fiber light source to guide nasogastric/orogastric tube insertion in neonates

**DOI:** 10.1038/s41372-023-01668-7

**Published:** 2023-04-05

**Authors:** Jumpei Kuroda, Kaoru Okazaki

**Affiliations:** Department of Neonatology, Tokyo Metropolitan Children’s Medical Center, Tokyo, Japan

**Keywords:** Paediatrics, Outcomes research, Preventive medicine, Medical imaging

Among hospitalized infants and children, the frequency of nasogastric tube (NGT) or orogastric tube (OGT) placements is highest in neonatal intensive care units [1]. NGT or OGT placement is a blind procedure. Therefore, it is not known whether the tip is positioned in the stomach after insertion. The NGT/OGT tip position is typically confirmed by methods including ultrasonography, auscultation, the pH of aspirate, and capnography [2]. However, there are concerns about most of these methods and their accuracy in a neonate, even x-ray. Moreover, frequent use of x-ray results in cumulative radiation doses in infants. The New Opportunities for Verification of Enteral tube Location project recommends pH measurement as the best practice for NGT placement verification in children [3]. This group proposed the need for product development to allow for placement verification and reverification in real time for the duration of NGT use [3]. Hirano et al. reported that using a light-emitting diode source and fiber (LED-SF) could confirm NGT tip positioning safely and simply in adults under general anesthesia [4]. LED-SF enabled NGT/OGT insertion into the stomach while viewing a red LED light at the tip of NGT/OGT without the need for a confirmatory x-ray. The specific aim of this study was to determine whether using LED-SF confirmed NGT/OGT tip in the stomach in neonates and to document the safety of this technology.

## Methods

**Fig. 1:**
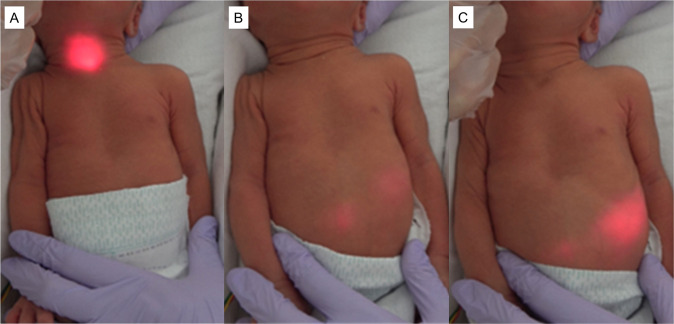
The red light emitted from the tip of the optical fiber was observable through the skin. The red light emitted by the LED was visible as it passed through the mouth and throat (**A**). Upon entering the stomach, the LED light illuminated the entire organ (**B**) but contracted to a red dot with advancement (**C**).

The prospective, observational cohort study examined routine NGT/OGT replacements conducted in ten infants at a level III NICU (Supplementary Table 1). Consent to participate was obtained from the subjects’ parents. The study protocol was approved by the Institutional Review Board of Tokyo Metropolitan Children’s Medical Center [2022b-32]. Informed parental consent was obtained for all patients. The LED-SF consisted of plastic optical fibers capable of lighting the tip as a red LED light. The LED-SF was first inserted into the NGT/OGT. Movement of the LED-SF tip from the NGT/OGT tip was prevented using a slide stopper, which fixed the position of the LED-SF tip at <1 cm from the NGT/OGT tip. NGT and OGT were used in nine and one neonates, respectively. The red light emitted by the LED was visible as it passed through the mouth and throat but was not visible in the esophagus (Fig. 1A). Upon entering the stomach, the LED light illuminated the entire organ (Fig. 1B) but contracted to a red dot with advancement (Fig. 1C). Based on the internal diameter of the NGT/OGT, a 0.5-mm diameter fiber (*n* = 7) or a 0.75 mm fiber (*n* = 3) was selected.

## Results

The LED-SF guided NGT/OGT tip to the correct position in the seven neonates and the position required slight adjustment in the three neonates (Supplementary Table 1). In the latter, NGT insertion was inappropriately paused when the LED illuminated the entire stomach (case 8). In the remaining two cases, the GT positioning required an adjustment of 0.5 or 1.0 cm. No adverse or safety events were observed during any procedure. No technical problems were encountered with its use, such as fiber breakage or light source leakage.

## Discussion

This is the first report in the literature demonstrating the safe and accurate NGT/OGT insertion using an LED-SF. In this study, the NGT/OGT tip was clearly visible as a red dot in the stomach of preterm or term neonates.

Perforations or misplacement related to the insertion of NGT/OGT in the stomach or esophagus are serious complications with mortality rates of up to 25.8% [5]. If perforation happened, the LED-SF would be expected to light not only the stomach but also the entire abdomen. In particular, esophageal perforation is an important and serious complication with a prevalence of (0.05%) in preterm infants with birth weight <1500 g and/or with gestational age ≤32 weeks. In this study, the insertion method of NGT/OGT was similar to the usual method and would not be expected to increase or decrease the risk of perforation. However, using LED-SF, the red LED light informed that the NGT/OGT tip safely passed the throat and correctly entered into the stomach without an x-ray. This is the strongest advantage. Furthermore, the frequency of x-ray may be less. The small sample size is our study limitation.

## Conclusion

We have demonstrated the safety and efficacy of an LED-SF to verify NGT/OGT placement in neonates. This technology gives staff real-time information about the movement of the tube tip during placement. Future studies in a larger cohort are necessary to validate the results derived in this pilot study and to demonstrate the feasibility of widespread use of LED-SF in neonates who require an NGT or OGT.

## Supplementary information

Supplementary Table 1
