# Supplementary material for: Pilot study using an optical fiber light source to guide nasogastric/orogastric tube insertion in neonates
Source: J Perinatol. 2023 Apr 5;43(9):1179–80. doi: 10.1038/s41372-023-01668-7 (PMC10497405; doi:10.1038/s41372-023-01668-7)
Supplement: Supplementary file 1 — Supplementary Table 1 [file 41372_2023_1668_MOESM1_ESM.docx]

Supplementary Table 1. Patient characteristics and findings.

Each insertion target depth was calculated using the formula commonly used in Japan: current height × 0.2 + 9 cm (NGT) or current height × 0.2 + 8 cm (OGT). cGA, corrected gestational age; ID, inner diameter.

| Data on enrollment | | | | | | | | | | |
| --- | --- | --- | --- | --- | --- | --- | --- | --- | --- | --- |
| Subject No. | 1 | 2 | 3 | 4 | 5 | 6 | 7 | 8 | 9 | 10 |
| Age [days] | 59 | 8 | 6 | 7 | 3 | 10 | 8 | 8 | 82 | 80 |
| cGA [weeks] | 42.1 | 34.9 | 35.6 | 36.3 | 37.9 | 35.3 | 38.7 | 34.9 | 39.3 | 39 |
| Weight [g] | 2350 | 1799 | 1807 | 2290 | 3123 | 1994 | 2729 | 1596 | 2014 | 2216 |
| Hight [cm] | 42.5 | 44.4 | 44.5 | 44.5 | 50 | 42.5 | 46.2 | 42 | 39 | 40.5 |
| Fiber ID [mm] | 0.50 | 0.50 | 0.50 | 0.50 | 0.75 | 0.75 | 0.75 | 0.50 | 0.50 | 0.50 |
| Insertion [cm] | 18.0 | 17.0 | 17.5 | 18.0 | 19.5 | 17.5 | 19.5 | 17.0 | 17.0 | 15.5 |
| Ajustment [cm] | 0.0 | 0.0 | 0.0 | 0.0 | 0.0 | 0.0 | 0.0 | -0.5 | 0.5 | 1.0 |
